# Identification of differentially regulated proteins in a patient with Leber's Congenital Amaurosis – a proteomic study

**DOI:** 10.1186/1477-5956-5-5

**Published:** 2007-02-27

**Authors:** Henrik Vorum, Morten Østergaard, Greg E Rice, Bent Honoré, Toke Bek

**Affiliations:** 1 Department of Ophthalmology, Aarhus University Hospital, Nørrebrogade 44, DK-8000 Aarhus C, Denmark; 2 Department of Medical Biochemistry, University of Aarhus, Denmark; 3 Translational Proteomics, The Baker Heart Research Institute, Melbourne, Australia

## Abstract

**Background:**

To identify the pattern of protein expression in the retina from a patient with Leber's Congenital Amaurosis (LCA) secondary to a mutation in the *AIPL1 *gene. The retina from one eye of a patient with LCA and 7 control eyes were studied. The tissue was subjected to high resolution two-dimensional gel electrophoresis, image analysis and mass spectrometry, in an effort to identify differentially regulated proteins.

**Results:**

In the LCA retina seven protein spots were differentially expressed. Six proteins were significantly up-regulated of which three could be identified as: αA-crystallin, triosephophate isomerase, and an N-terminal fragment of the β-chain of ATP synthase. One protein spot that was down-regulated in the LCA retina was identified as a C-terminal fragment of β-tubulin.

**Conclusion:**

Retinal tissue in LCA is characterised by an up-regulation of αA-crystallin, triosephosphate isomerase, and ATP synthase (β-chain fragment) and down-regulation of a fragment of β-tubulin. These proteins/protein fragments may play a crucial role for the retinal degeneration processes in LCA and other retinal dystrophies.

## Background

In 1869 Leber described a disorder associated with congenital amaurosis, nystagmus, and the oculodigital sign that appeared to be a variety of retinitis pigmentosa. This disorder, now referred to as Leber's congenital amaurosis (LCA), is a group of autosomal recessive dystrophies with a heterogenous clinical and genetic background [1]. To date, mutations of seven genes have been reported to be implicated in the disease: *RetGC1 *[2,3], *RPE65 *[4,5], *CRX *[6], *AIPL1 *[7,8], *LRAT *[9], *CRB1 *[10], and *RPGRIP *[11]. In addition, two other loci may be involved: *LCA3 *on 14q24 [12] and *LCA5 *on 6q11-16 [13].

LCA occurs at an incidence of 3/100,000 newborns and currently no treatment is available. The pathophysiology of LCA is unknown, however, histological data are consistent with abnormal development of photoreceptor cells in the retina and extreme premature degeneration of retinal cells [8,14-16]. It is conceivable that analysis of the differential expression of retinal proteins in LCA may provide further insight into the pathophysiology of the disease. We, therefore, performed proteomic analysis [17] of retinal tissue in 7 normal persons and one patient with LCA due to a mutation in the AIPL1 gene [7,8]. APL1 (aryl hydrocarbon receptor-interacting protein-like 1) is a member of the FK-506-binding protein family that is specifically expressed in retinal photoreceptors. The possible significance of the differential expression of proteins in the LCA patient as compared to the normal persons is discussed.

## Results

**Figure 1:**
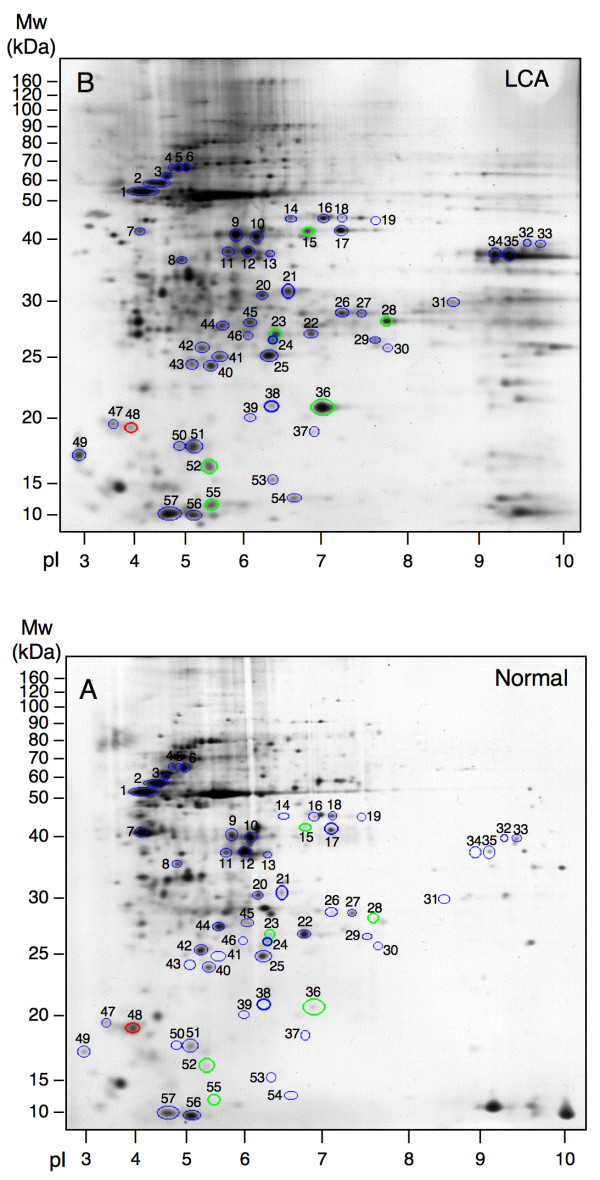
Representative 2D gels from LCA retina (**A**) and from normal retina (**B**). Fifty seven silver-stained spots (encircled) were analysed. The 6 proteins found to be significantly up-regulated (p < 0.05) are marked by green circles. The single significantly down-regulated protein (p < 0.05) is encircled in red. The remaining 50 protein spots included in the analysis are marked by blue circles.

**Figure 2:**
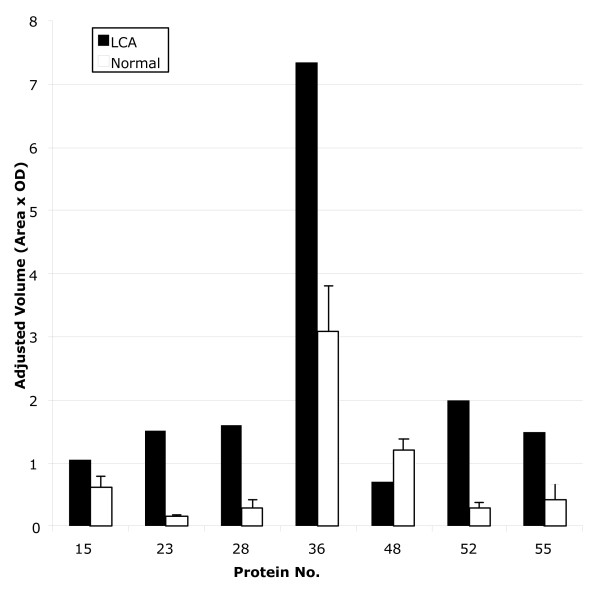
Histograms of adjusted protein spot volumes of the 7 spots that were found to be differentially regulated. LCA: Actual volume. Normal: Mean volume ± SD.

Representative examples of the retinal protein expression pattern as revealed by 2D-PAGE are shown in figure 1 for the LCA retina and the normal retina. The overall protein expression profiles were similar. Fifty seven well-separated and clearly focused protein spots were included in the analysis. Volumes of each of the 57 spots were calculated. Seven protein spots were found to be differentially expressed (figure 2) when calculated as described in the Methods section. Six protein spots from the LCA gel were significantly up-regulated by a factor of 1.7 – 9.8 (p < 0.05) and one protein spot was significantly down-regulated by a factor of 1.7 (p < 0.05) (Table 1).

**Table 1:** Comparison of adjusted spot volumes of the 7 protein spots that were found to be differently regulated.

**Protein No.**	**Adjusted spot volume (OD × Area)**		p-value*	**Relative protein levels in LCA to normal**
			
	**LCA (n = 1)**	**Normal (n = 7) Mean ± SD**		
15	1.066	0.616 ± 0.175	0.042	1.73
23	1.505	0.154 ± 0.016	<0.0001	9.78
28	1.600	0.290 ± 0.116	<0.0001	5.52
36	7.345	3.073 ± 0.719	0.001	2.39
48	0.699	1.192 ± 0.173	0.029	0.59
52	2.000	0.291 ± 0.089	<0.0001	6.88
55	1.498	0.423 ± 0.239	0.004	3.54

*The p-value was calculated by comparing the single observation from LCA with the range observed in the normal retina using the t-distribution (see Materials and Methods).

Using mass spectrometry 3 of the up-regulated proteins could be identified as: αA-crystallin, triosephophate isomerase, and an N-terminal fragment of ATP synthase. Three of the up-regulated proteins in the LCA retina could not be identified. The down-regulated protein was identified as a C-terminal fragment of β-tubulin. The sequence coverage of the identified proteins ranged from 11% to 33% (Table 2).

**Table 2:** Identification of differentially regulated proteins in LCA retina

**Protein No.**	**Identification**	**Identified peptides**	**Coverage %**	**pI**	**Mr (kDa)**	**Function**	**Swiss-Prot Accession Number**
15	ND*						
23	ND						
28	Triosephosphate isomerase	IAVAAQNCYK (59–68) TATPQQAQEVHEK (175–187) TATPQQAQEVHEKLR (175–189) SNVSDAVAQSTR (194–205) IIYGGSVTGATCK (206–218)	20	7.1	26.89	Enzyme	P60174
36	αA-crystallin	RTLGPFYPSR (12–21) TLGPFYPSR (13–21) TVLDSGISEVR (55–65) QDDHGYISR (104–112) IQTGLDATHAER (146–157) AIPVSREEKPTSAPSS (158–173)	33	5.8	20.01	Chaperone	P02489
48	β-tubulin (C-terminal fragment)	NSSYFVEWIPNNVK (119–132) TAVCDIPPR (133–141) RISEQFTAMFR (162–172) ISEQFTAMFRR (163–173)	11	4.9	26.03	Cytoskeletal	P68371
52	ATP synthase β-subunit precursor (N-terminal fragment)	LVLEVAQHLGESTVR (95–109) TIAMDGTEGLVR (110–121) VLDSGAPIKIPVGPETLGR (125–143) IMNVIGEPIDER (144–155)	11	5.3	56.54	Oxidative phosphorylation	P06576
55	ND						

* ND, not determined.

† Spectra obtained by MALDI-TOF were used to search the Swiss-Prot databases. The peptides given were in agreement with measured masses. Number in parentheses indicate the position of the peptide in the protein.

**Figure 3:**
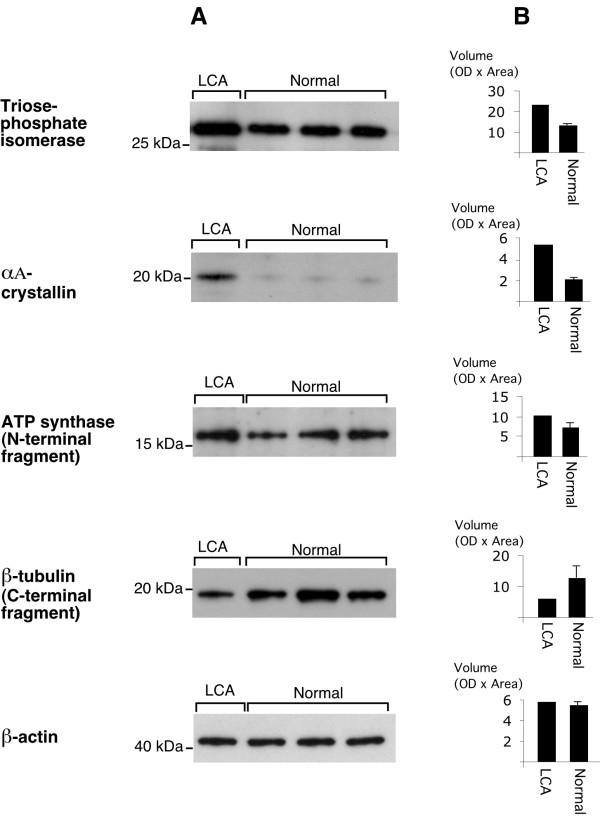
Western blot analysis of retina from the LCA patient and 3 normal individuals. The blots in panel **A **were incubated with antibodies against the appropriate antigens. β-actin was used as a loading control. The histograms in panel **B **represent densitometry (OD × Area) of the blots. (LCA: Actual volume. Normal: Mean volume ± SD).

In order to verify the quantitation of spot density on 2D gels, we also analysed retinal samples by 1D Western blotting using commercially available antibodies. As seen from the Western blots (figure 3A) it was possible qualitatively to verify the molecular weights as well as the differential expression of each of the four proteins/protein fragments. β-actin was used as a loading control. In addition, quantitative densitometry for the immune reactions (figure 3B) was also carried out. Estimated from the 2D gels αA-crystallin was up-regulated by a factor 2.39 (table 1) and from the Western blot by a factor 2.74, Triosephosphate isomerase by a factor 5.52 (2D gels) and 1.73 (Western blot), ATP synthase β-subunit by a factor 6.88 (2D gels) and 1.40 (Western blot), whereas β-tubulin was down-regulated by a factor 0.59 (2D gels) *versus *0.47 (Western blot). Using these two completely different methods, data from the 2D gels *versus *data from Western blots demonstrated exactly the quantitative trend for each of the proteins in question.

Scleral tissue was used as a control tissue not known to be involved in the pathology of LCA, and did not show any significant difference in protein expression between the LCA eye and one normal eye (not shown).

## Discussion

Leber's congenital amaurosis is an ocular syndrome that includes retinal dystrophy with lesions characteristic of retinitis pigmentosa. To date at least seven genes have been reported to be implicated in the disease, however, it is unknown how the affected genes as well as other genes are expressed to develop the dystrophic changes. Therefore, mapping of the protein expression pattern of the diseased LCA retina may contribute to further understanding the pathogenesis of the disease.

The aim of this study, was to identify LCA-associated differential expression of retinal proteins using 2D-PAGE. Retinal proteins from one patient and 7 normal individuals were compared. Inspected by eye, the overall 2D-PAGE profile from the LCA patient was similar to that of the normal individuals. Fifty seven protein spots that were found to be present in all gels were analysed. In order to estimate the quantitative similarity of the 2D profiles, the coefficient of variation was calculated to 24.4% (6.3% to 38.3%) for the normal retinas in this biological material.

Disease-associated differential expression of proteins, however, was identified in retinal but not scleral tissue used as a control tissue not known to be involved in the pathology of LCA. In LCA retina, the expression of 7 protein spots was altered by at least 1.5-fold when compared to normal retina. Six protein spots were up-regulated and one down-regulated in LCA retinal tissue.

Differentially expressed proteins were subsequently excised from the gels for mass spectrometry identification. By this process we identified 3 up-regulated proteins as, αA-crystallin, triosephosphate isomerase, and an N-terminal fragment of ATP synthase. The down-regulated protein was identified as a C-terminal fragment of β-tubulin. All these proteins are encoded from nuclear genes except for ATP synthase which is encoded from the mitochondrion genome. Three proteins unfortunately could not be identified by mass spectrometry. Several reasons may account for this, *e.g*. low quantities of protein, peptide modification due to the silver staining procedure and low molecular mass proteins generally produce only few peptides for fingerprinting.

### αA-crystallin

The α-crystallins belong to the small heat shock protein family and make up more than 90% of the total dry mass of the lens [18], and thus contribute to the refractive index of this structure. The lens αA-crystallin is also expressed in other eye tissues such as the retinal Müller-cells and the photoreceptors, and may have similar optical functions in these cell types. In addition, αA-crystallin possesses a chaperone-like function and is involved in the first steps of protein synthesis by binding to the unfolded proteins to avoid non-specific aggregation. The involvement of αA-crystallin in human disease has recently been reported by the observation of a single missense mutation, R116C, which is associated with cataract formation [19]. The role of αA-crystallin up-regulation in LCA is unknown, but could be related to a stressful condition in the retina during the disease process.

### Triosephosphate isomerase

Triosephosphate isomerase is a glycolytic enzyme catalysing the interconversion of D-glyceraldehyde 3 phosphate to dihydroxyacetone phosphate (DHAP) in the glycolytic pathway. Glucose is the major energy source in the brain and the retina. A strong up-regulation of a glycolytic enzyme might be associated with an increased glucose metabolism in the retina of diseased individuals. On the other hand, if the LCA retina is not particularly metabolic active the up-regulation could be explained by other as yet unknown functions of triosephosphate isomerase. As an example another key glycolytic enzyme, α-enolase, is in fact a multifunctional protein that possesses additional extracellular functions besides the well-known intracellular function [20].

### ATP synthase β-subunit (N-terminal fragment)

The ATP synthase is the only protein complex found in the present study that is encoded by the mitochondrion genome. The complex consists of a soluble region, F_1_, and a membrane bound region, F_0_. Functionally, the synthase may be divided into two counter-rotating parts: the rotor consisting of subunits γ and ε and the cylindrical c subunits while the stator consists of the α_3_β_3 _hexamer connected via the δ and b_2 _subunits to the a subunit. A detailed review of the structural and functional properties of the ATP synthase has recently been published [21]. The a subunit is part of the stator, localized in the membrane and serves as a channel through which ions may enter and bind to the rotor. We find that an N-terminal fragment (the extremity of the protein) of the β subunit is up-regulated in the retina of patients with LCA. This might indicate an increased degradation of the β subunit of ATP synthase. LCA has previously been associated with mutations at nucleotide 9101 in the ATP synthase of the mitochondrion genome [22] as well as with mutation in another mitochondrial gene, NADH dehydrogenase subunit 4 [23].

### β-tubulin, (C-terminal fragment)

β-tubulin (55 kDa) belongs to the tubulin superfamily that to date consists of seven distinct members, α-, β-, γ-, δ-, ε-, ζ-, and η-tubulin. The tubulins constitute one of the major components of the cytoskeleton of eukaryotic cells and are involved in a variety of processes such as cell division, ciliary function and intracellular transport. β-tubulin forms a heterodimer with α-tubulin that constitutes the basic building block of microtubules and together with γ-tubulin appear to be present in all eukaryotic cells, sometimes the only tubulin variety present [24]. The fact that we find a 20 kDa C-terminal fragment (the extremity of the protein) of the protein to be down-regulated may indicate decreased cleavage of β-tubulin, but the significance of this finding otherwise remains to be elucidated.

## Conclusion

In conclusion, we have by proteomic analysis found significant perturbations in the expression of seven retinal proteins in Leber's congenital amaurosis. There is no apparent direct functional link between the observed proteins indicating that a complex series of molecular events are involved in the pathogenesis of the disease. Some of the differentially regulated proteins could be categorized as stress regulated proteins and proteins involved in the formation of energy compounds. It might be that LCA disease leaves the retina in a "stressful" condition, up-regulating chaperones and with changes in metabolism and energy consumption.

In this study, it is not possible to negate unequivocally a contribution of age-related effects on retinal protein expression. In a previous study of rat retinal proteins, however, no age-related effects on proteins displayed by 2D-PAGE were identified [25]. In addition, the tissue specificity of differential expression of proteins further supports the contention that the observed differences are disease and not age related. An analysis of the effects of ageing on retina protein expression would be required to resolve this issue.

This study emphasizes that although the identification of mutations in the human genome is a precondition for understanding inherited diseases, a full understanding of the pathophysiology of these diseases requires a detailed mapping of the structural and metabolic consequences of the mutations. This may be a laborious and complex challenge in ocular diseases in general and Leber's congenital amaurosis in particular.

## Methods

### Clinical case

The patient was a son of unrelated parents, and his four years older sister had LCA diagnosed at the age of one year. At the age of five months he underwent a clinical examination which showed no light perception in either of the eyes. Ophthalmoscopy revealed greyish atrophic retinas, and electroretinography showed extinguished signals in both eyes. At the age of 22 years the patient was admitted because of constant headache and pain in both eyes which he attempted to relieve by poking the eyes. The clinical examination showed bilateral nystagmus and no light perception. Slit lamp examination showed bilateral keratoconus and posterior polar cataract. Ophthalmoscopy gave a slightly blurred view of the fundi. The far periphery was avascular and with hyperpigmentations dispersed in the retina. On the patient's request both eyes were enucleated, which relieved the pain immediately.

### Control cases

Seven normal individuals 54–81 years old were included. Five cases were autopsies without known eye diseases. In the last two cases an orbital exenteration in combination with enucleation were performed after the persons gradually developed proptosis and diplopia due to tumors in the orbit not involving the eyes. In alle cases slit lamb examination and ophthalmoscopy showed a normal fundus with no inflammation or sheared stress. When compared, no differences were seen in the protein profiles obtained from the 7 normal individuals.

### Tissue processing

Processing of ocular tissue for proteomic analysis in our laboratory has been approved by the local scientific ethics committee. Informed consent for the investigations was obtained from both the LCA patient and the normal individuals in accordance with the provision of the Declaration of Helsinki. Immediately after enucleation, the eyes were divided by a section along the horizontal meridian. In both the LCA patient and the controls a 4 × 4 mm piece of retina was excised from the section line to represent equal parts and were prepared for two-dimensional gel electrophoresis as previously described [27]. From the LCA patient and from a single control a 4 × 4 mm piece of sclera was also excised and prepared for two-dimensional gel electrophoresis.

### Two-dimensional Polyacrylamide Gel Electrophoresis (2D-PAGE)

The first dimension isoelectric focusing (IEF) was performed using non-linear pH 3-10NL IPG strips (Amersham Biosciences). Rehydration of the IPG strip was performed using the Immobiline DryStrip Reswelling Tray (Amersham Biosciences). The IPG strip was rehydrated for 20 h at room temperature in 400 μl protein sample containing approximately 100 μg of protein. IEF was carried out on a Multiphor II electrophoresis unit (Amersham Biosciences) at 500 V for 0.01 h, 500 V for 3 h, 3500 V for 5 h, and 3500 V for 20 h in a gradient mode at 17°C using a MultiTemp III Thermostatic Circulator (Amersham Pharmacia Biotech AB). Prior to the second dimension SDS-PAGE, the IPG strip was equilibrated for 2 times 15 min under gentle agitation in 10 ml of equilibration solution (0.1%, Tris-HCl, pH 6.8, 5.5 M Urea, 0.3% Glycerol, 0.035 M SDS, and 0.065 M DTT). Instead of DTT, iodoacetamide (14 mM) and traces of bromophenol blue were added to make the second equilibration solution. For the second dimension the equilibrated IPG strip was transferred to a home made polyacrylamide gel (5% stacking and 15% resolving). The electrophoresis for the second dimension was performed vertically at a maximum voltage of 50 V, 5 mA for approximately 20 h in custom made equipment previously designed and described [27]. With the employed technique proteins were separated in the pI range between 3 and 10 (first dimension) and in the molecular mass range between 10 and 160 kDa (second dimension).

### Silver staining

The gels were visualised by silver staining using the protocol of Vorum optimised for high sensitivity protein identification by mass spectrometry [28]. Firstly, the gel was fixed in 50% (v/v) methanol, 12% (v/v) acetic acid, 0.0185% (v/v) formaldehyde overnight or at least for 1 h, then washed 3 times for 20 min in 35% (v/v) ethanol and pre-treated for 1 min in 0.02% (w/v) Na_2_S_2_O_3_, 5H_2_O, rinsed in water 2 times for 3 min, stained in 0.2% (w/v) AgNO_3_, 0.028% (v/v) formaldehyde for 20 min, rinsed with water 2 times for 20 s and developed in development solution: 6% (w/v) Na_2_CO_3_, 0.0185% (v/v) formaldehyde, 0.0004% (w/v) Na_2_S_2_O_3_, 5H_2_O, for approximately 3 min. Finally, development was stopped in stop solution: 50% (v/v) methanol, 12% (v/v) acetic acid. The gels were mounted between two sheets of cellophane and dried.

### Analysis of two-dimensional gels

Initially proteins from one normal retina and one LCA retina were separated by 2D-PAGE. The gels were silver stained and scanned using a Bio-Rad GS-710 Calibrated Imaging Densitometer (Bio-Rad Laboratories, Hercules, CA, USA). The spots were analysed using Bio-Rad Multi-Analyst (V.1.02) software, which designates a volume (spot area times density) to each spot, proportional to the amount of protein. The volume of each spot in the gel was converted to an adjusted volume normalised to the total amount of protein in the gel. In an effort to identify differentially expressed proteins, we tested whether the volumes of the protein spots in the LCA gel were different from the corresponding spots in the normal gel. In this pilot study 8 proteins were found to be differentially expressed. We then extended the study by including 6 additional 2D gels from normal retinas, in an effort to confirm the differential expression of the 8 proteins. After re-analysis only 7 proteins were found to be significant differentially expressed.

The difficulty in obtaining adequate samples for analysis of this very rare human disease makes statistical analysis complicated, and due to the very limited data set such analysis should be interpreted with caution. However, we approached a calculation of the probability that a single observation (the volume of a given spot in the LCA gel) was within the range of the volumes of the same spot in the normal gels by using the t-distribution: t = (|μ-x|)/S; where μ is the mean of the spot volumes in the normal gels, x is the single observation in the LCA gel and S it the standard deviation of μ. From the t-values obtained, we calculated the two-tailed p-values. Spots that possessed p-values below 0.05 were considered as significant and selected for identification by MALDI-TOF mass spectrometry.

### Identification of proteins by mass spectrometry

Gels containing protein spots selected for identification were re-swelled in water. The cellophane sheets were peeled off so that the spots could be excised from the gels. Proteins were subjected to trypsin digestion and subsequent identification by MALDI-TOF mass fingerprinting (MS), performed as a service by Alphalyse A/S (Odense, Denmark). The MS data was submitted to the ProFound – Peptide Mapping facility [29].

### Western blotting

The protein concentrations were measured with a Non-Interfering Protein Assay (Geno Technology, Inc., St. Louis, MO, USA). Ten μg of each samples were loaded and run on 4–20% gradient polyacrylamide gels. After transfer by electroelution to nitrocellulose Hybond C-extra membranes, blots were blocked with 5% milk, 5% fetal calf serum, and 0.05% Tween-20 in 80 mM Na_2_HPO_4_, 20 mM NaH_2_PO_4_, 100 mM NaCl, pH 7.5 (PBS-T), and incubated overnight at 4°C with anti-triosephosphate isomerase (Everest Biotech, Oxfordshire, UK) diluted 1:1,000, anti-αA-crystallin (Acris Antibodies, Hiddenhausen, Germany) diluted 1:1,000, anti-β-tubulin (Abcam, Cambridge, UK) diluted 1:1,000, and anti-ATP synthase subunit β (Abcam, Cambridge, UK) diluted 1:500, anti-β-actin (Abcam, Cambridge, UK) diluted 1:100. The labeling was visualised with horseradish peroxidase-conjugated secondary antibodies (P217, P260 or P449, DAKO, Glostrup Denmark, diluted 1:1,000 to 1:5,000) by using the enhanced chemiluminescence system (Amersham International).

## Competing interests

The author(s) declare that they have no competing interests.

## Authors' contributions

HV carried out tissue processing, 2D-PAGE and silver staining, western blotting, conceived of the study, participated in design of the study and drafted the manuscript. MØ carried out 2D gel- and protein analysis, and helped to draft the manuscript. GR helped to draft the manuscript. BH carried out protein analysis and helped to draft the manuscript. TB participated in design of the study and helped to draft the manuscript. All authors read and approved the final manuscript.
